# Association between monocyte-to-lymphocyte ratio and tuberculin skin test positivity in HIV-positive adults

**DOI:** 10.1371/journal.pone.0253907

**Published:** 2021-07-16

**Authors:** Eva Van Ginderdeuren, Jean Bassett, Colleen F. Hanrahan, Annelies Van Rie

**Affiliations:** 1 Witkoppen Clinic, Johannesburg, South Africa; 2 Faculty of Medicine and Health Sciences, Family Medicine and Population Health, University of Antwerp, Antwerp, Belgium; 3 Epidemiology, Johns Hopkins Bloomberg School of Public Health, Baltimore, Maryland, United States of America; Brigham and Women’s Hospital, UNITED STATES

## Abstract

**Background:**

The tuberculin skin test (TST) identifies individuals at high risk of developing tuberculosis (TB) but poses many challenges. The blood monocyte-to-lymphocyte ratio (MLR) could be an alternative, as extremes in MLR have been associated with increased risk of TB disease.

**Methods:**

At a primary care clinic in Johannesburg, a differential white blood cell count and TST was performed in adults starting antiretroviral treatment (ART) without symptoms suggestive of active TB.

**Results:**

Of 259 participants, 171 had valid results of whom 30% (51/171) were TST positive and the median MLR was 0.18 (IQR 0.13–0.28). The MLR distribution differed between CD4 count categories (p < 0.01), with a broader range of values in TST negative participants with a low CD4 count (≤ 250 cells/mm^3^), likely reflecting HIV immunosuppression. MLR was associated with a positive TST (OR 0.78 per 0.1 increase, 95% CI 0.59, 0.97) in bivariate analysis but not in multivariate regression analysis (aOR 0.83 for every 0.1 increase, 95% CI 0.60, 1.08).

**Conclusion:**

In ART-naïve adults without symptoms suggestive of active TB, MLR was not independently associated with TST positivity and is thus unlikely to be a useful alternative to TST. Future research should focus on development of a cheap, simple and accurate biomarker to identify those people benefiting most from preventive TB therapy.

## Introduction

TB remains a global public health threat, with 10 million new tuberculosis (TB) cases and 1.5 million TB deaths annually worldwide [1]. Among people living with HIV, TB remains an important cause of morbidity and mortality with almost a million TB cases and a quarter million TB deaths in 2018 despite the impressive global roll-out of antiretroviral therapy (ART) [1]. Investing in effective tuberculosis preventive treatment (TPT) continues to be an essential component of the TB elimination strategy [2]. One of the greatest challenges facing TB prevention programs is the identification of people at highest risk of progression to active TB in order to maximize their benefit and limit the burden of a TPT course.

For decades, the tuberculin skin test (TST), a surrogate of infection with *Mycobacterium tuberculosis*, has been widely used to identify people at increased risk of developing TB disease [3]. Important barriers to the use of TST include its cost, logistics with need for maintenance of the cold chain (i.e. maintaining control of temperature throughout the supply chain), impact on human resources, and requirement for patients to return for TST reading within 48–72 hours [4,5]. Interferon-Gamma Release Assays (IGRAs), an alternative in vitro diagnostic to the TST [6], is more costly and technically complex than the TST, and only recommended by the World Health Organization (WHO) to replace the TST in low- and middle-income countries subject to availability and affordability [5]. An important challenge to effective use of either TST or IGRA is the reduced sensitivity in ART-naïve individuals living with HIV due to HIV immunosuppression [7].

There is thus a need for a cheap, simple and reliable method to identify those people at highest risk of developing TB. A monocyte-to-lymphocyte ratio (MLR) has been shown to predict an increased risk for TB disease in adults starting ART [8], therefore the MLR may be an alternative to the TST, as a full blood count is cheap and widely available in almost all laboratories worldwide. The association between TST and MLR has however not yet been assessed. We conducted a cross-sectional study to evaluate the association between MLR and TST positivity in ART-naïve individuals presenting for HIV care.

## Methods

The study was performed at the Witkoppen primary care clinic servicing Diepsloot, an urban, densely populated, poor informal settlement of approximately 12 km^2^, with an estimated population of 138,329 in northern Johannesburg [9]. From November 2016 to September 2017, ART-naïve adults (≥ 18 years) without a diagnosis of active TB or without symptoms suggestive of active TB (cough, fever > 2 weeks, weight loss, night sweats) who had a TST done as part of routine pre-ART assessment were eligible for study participation and invited to participate in the study. At enrolment, a differential blood count to determine monocyte and lymphocyte count was requested in addition to the CD4 count and other routine pre-ART assessment. Blood samples were analysed by the National Health Laboratory Service (NHLS) according to routine standard of care. Participants were asked to return to the clinic for TST reading within 48–72 hours. TST was read blinded to the laboratory results, and a TST ≥ 5 mm was considered positive [10]. Clinic files and the electronic NHLS database were reviewed to document age, gender, pregnancy status, date of HIV diagnosis, TST results (mm), history of tuberculosis treatment, CD4 count (cells/mm^3^), absolute monocyte count (10^9^/L), and absolute lymphocyte count (10^9^/L).

### Ethics statement

Ethical approval was obtained from the Health Research Ethics Committee of the University of the Witwatersrand (M150782). All participants gave written informed consent.

### Statistical methods

The MLR was calculated by dividing the absolute monocyte count by the absolute lymphocyte count. Participants´ characteristics were summarized using proportions and medians. Proportions were compared using Chi-Square or Fisher’s exact test; medians by Mann-Whitney U test or the Kruskal-Wallis test. To visualize the relationship between the TST results and MLR, we generated a scatterplot and calculated the Pearson correlation between quantitative TST results (in mm) and MLR. To assess the effect of CD4 count, we made violin plots of MLR and TST positivity stratified by CD4 count category (CD4 ≤ 250, 250 < CD4 ≤ 500, CD4 > 500 cells/mm^3^), and compared MLR results by CD4 count categories (Kruskal Wallis test) and by TST status (Mann-Whitney U test). To test for differences in dispersion, the Levene’s test was employed. The relationship between monocyte count and lymphocyte count, stratified by CD4 count categories, was also explored by scatterplots among TST positive and TST negative individuals. To test the hypothesis that MLR is associated with TST status, we performed logistic regression analysis to assess the association between the MLR and qualitative TST results (negative vs positive result), and expressed the strength of association using crude odds ratios (OR) and adjusted OR (aOR), and aOR stratified by CD4 count category, a possible effect modifier. For the multivariate analyses, correlated variables (|r| > 0.5) were removed to avoid multicollinearity, univariable prefiltering was not employed [11]. The goodness-of-fit of the logistic regression model was assessed using the Hosmer-Lemeshow test. To explore potential non-linearity in the association between MLR and qualitative TST results (negative vs positive result), spline regression was performed. All statistical tests were 2-sided, and the significance level used was 0.05. All statistical analyses were performed in R.

## Results

In total, 259 participants were enrolled. Two participants were excluded as they were diagnosed with extrapulmonary TB after enrolment. MLR values were available for 229 (89%) participants, of whom 171 (72%) had a valid TST result and were included for analysis (Tables 1 and S1). Median age of the 171 participants was 33 years (IQR 29–40), and 68% (n = 117) were female, of whom 18 were pregnant. Few (5/171, 3%) reported a previous episode of TB treatment. Median CD4 count was 263 cells/mm^3^ (IQR 151–436), 81 (47%) had a CD4 count ≤ 250 cells per mm^3^, 54 (32%) between 250 and 500 cells per mm^3^ and 36 (21%) had a CD4 cell count ≥ 500 cells per mm^3^. No significant differences in socio-demographics or laboratory results were observed between the 171 participants included in the analysis and the 88 participants excluded due to missing MLR and/or TST. Among the 88 excluded participants, the majority was female (56/88 or 64%), of whom 8 were pregnant. The median age was 32 years (IQR 27–37) and median CD4 count was 284 cells/mm^3^ (IQR 124–458).

**Table 1:** Patient characteristics by TST status of 171 ART-naïve adults presenting for HIV care at a primary clinic in northern Johannesburg.

Characteristic	TST positive (n = 51)	TST negative (n = 120)	p-value
Median age in years (IQR)	33 (29–39)	34 (28–40)	0.65
Female	34 (67%)	83 (69%)	0.75
Pregnant	8 (16%)	10 (8%)	0.15
Median BMI in kg/m^2^ (IQR)^*#*^	25 (23–30)	25 (22–29)	0.25
History TB treatment	2 (4%)	3 (3%)	0.64
New HIV diagnosis (same day as enrolment)	46 (90%)	110 (92%)	0.77
Most recent CD4 in cells/mm^3^ in categories*			< 0.001
CD4 ≤ 250 (n = 81)	13 (25%)	68 (57%)	
250 < CD4 ≤ 500 (n = 54)	18 (35%)	36 (30%)	
CD4 > 500 (n = 36)	20 (39%)	16 (13%)	

^#^BMI data missing in 1 participant with positive TST. TST = tuberculin skin test; ART = antiretroviral treatment; HIV = human immunodeficiency virus; IQR = interquartile range; BMI = body mass index; TB = tuberculosis; MLR = monocyte-to-lymphocyte ratio.

Overall, monocyte count ranged from 0.08 x 10^9^/L (below the 0.18 x 10^9^/L lower reference range for adults) to 1.31 x 10^9^/L (above the upper normal reference of 0.8 x 10^9^/L), with a median of 0.26 x 10^9^ cells/L (IQR 0.21–0.35) [12]. Lymphocyte count ranged from 0.14 x 10^9^/L (below the 1.0 lower reference range) to 4.82 x 10^9^/L (above the upper normal reference of 4.0), with a median of 1.52 x 10^9^/L (IQR 1.08–1.92) [12]. The MLR ranged from 0.04 to 1.14 with a median MLR of 0.18 (IQR 0.13–0.28).

**Fig 1:**
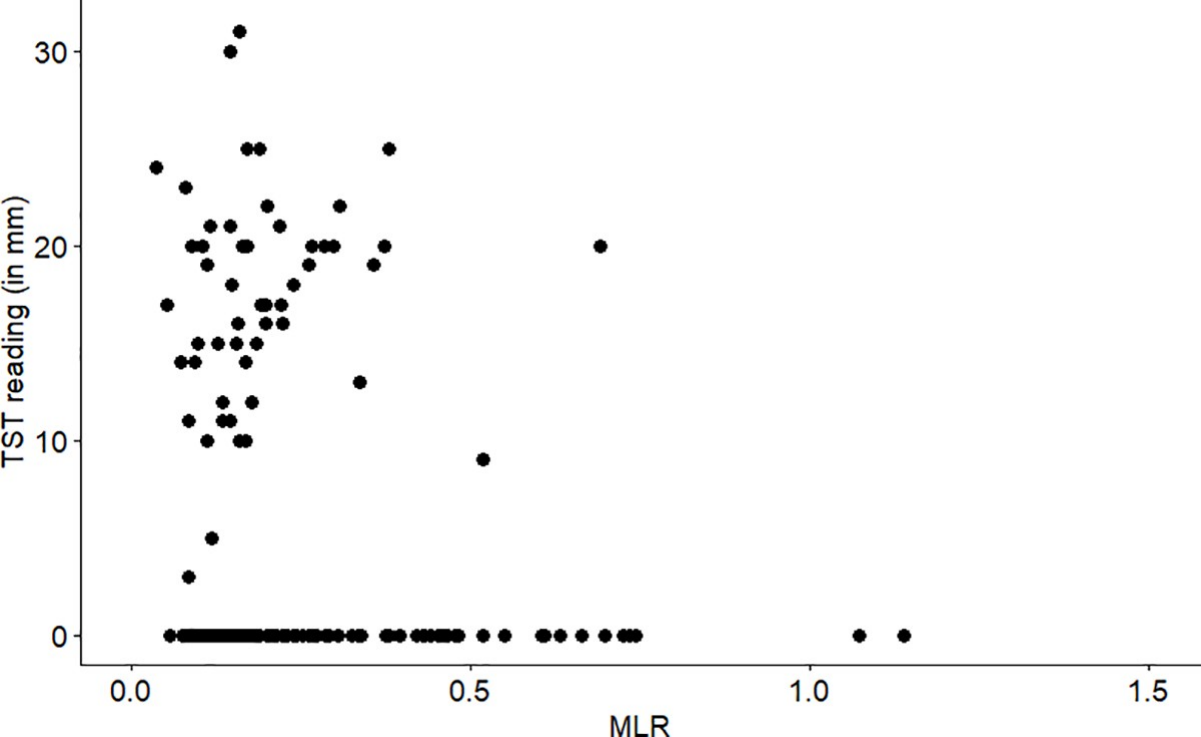
Scatterplot of MLR and quantitative TST values (in mm) among all 171 participants. MLR = monocyte-to-lymphocyte ratio; TST = tuberculin skin test.

Overall, 51 (30%) participants had a positive TST with a median diameter of 18 mm (IQR 14–20). The proportion of participants with a positive TST was higher among those with CD4 count > 250 cells/mm^3^ compared to those with CD4 count ≤ 250 cells/mm^3^ (42% or 38/90 vs 16% or 13/81, p < 0.001, Table 1). The median MLR did not differ by TST status (0.17 among TST positive participants vs 0.18 among TST negative participants, p = 0.11). The range of MLR was a bit narrower among participants with a positive TST than among participants with a negative TST (0.04 to 0.69 vs. 0.06 to 1.14, p = 0.04) (Table 1, Fig 1). Among those with a positive TST, there was not a linear association between TST size (in mm) and MLR (r 0.20, p = 0.18, n = 45) (Fig 1). In bivariate regression analysis (Table 2), participants with a positive TST were more likely to have a lower MLR [OR 0.78 (95% CI 0.59, 0.97) for every 0.1 unit increase in MLR], but this association was no longer significant in multivariate analysis [aOR 0.83 (95% CI 0.60, 1.08) for every 0.1 unit increase in MLR].

**Table 2:** Bivariate (crude OR) and multivariate logistic regression analysis (adjusted OR) of the relationship between MLR (per 0.1 unit increase) and qualitative TST results (negative vs positive result), overall and stratified by CD4 count category.

		Crude OR of MLR (95% CI)	aOR of MLR (95% CI)^¶^
Unstratified analysis		0.78 (0.59, 0.97)	0.83 (0.60, 1.08)
Stratified analysis	CD4 ≤ 250 cells/mm^3^	0.98 (0.36, 2.60)	0.95 (0.30, 2.87)
	250 < CD4 ≤ 500 cells/mm^3^	0.89 (0.55, 1.48)	0.87 (0.47, 1.72)
	CD4 > 500 cells/mm^3^	1.22 (0.89, 1.87)	1.35 (0.94, 2.23)

^¶^Adjusted for CD4 count category (only in unstratified analysis), age, sex, pregnancy status, BMI, history TB treatment, new HIV diagnosis. Monocytes and lymphocytes excluded in the multivariate analysis as there was a high correlation with MLR [monocytes (r = 0.52), lymphocytes (r = -0.55)]. OR = odds ratio; aOR = adjusted odds ratio; TST = tuberculin skin test; MLR = monocyte-to-lymphocyte ratio; CI = confidence interval.

**Fig 2:**
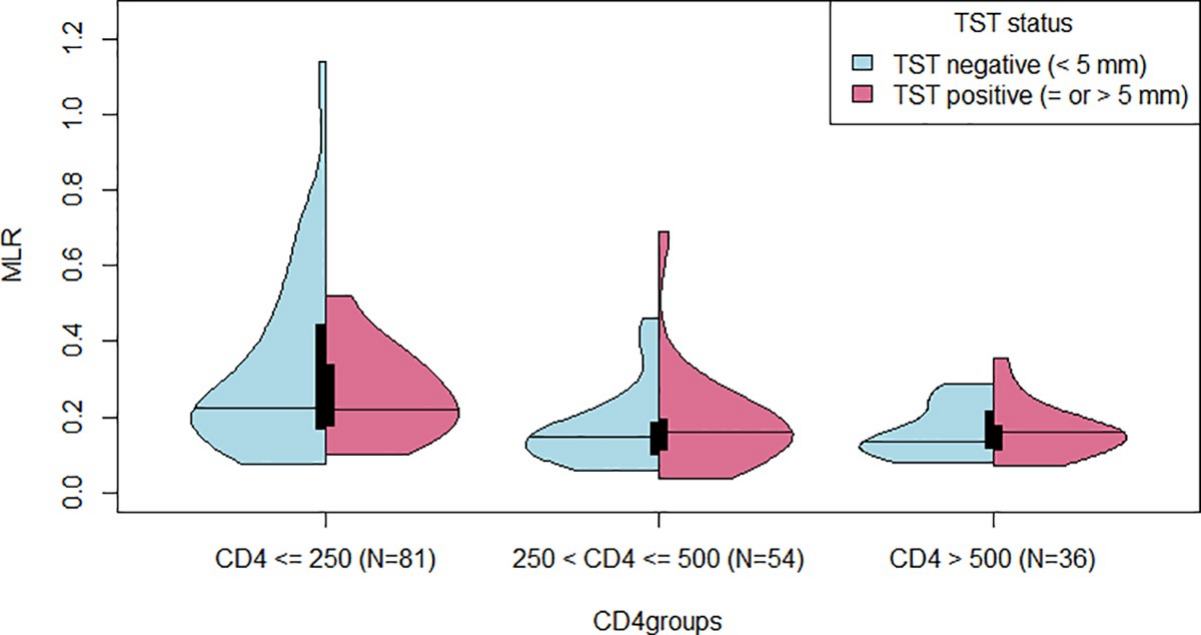
MLR stratified by TST status (negative vs positive TST result) and CD4 count category (≤ 250, 250 < CD4 ≤ 500, > 500 cells/mm^3^). The medians are displayed by horizontal lines and interquartile ranges by black boxes. MLR = monocyte-to-lymphocyte ratio; TST = tuberculin skin test.

A positive TST was strongly associated with CD4 count category, with increasing odds of a positive TST with higher CD4 count [OR 2.62 (95% CI 1.16, 6.04) for 250 < CD4 ≤ 500 and OR 6.54 (95% CI 2.75, 16.28) for CD4 > 500] (S1 Table). The median MLR differed by CD4 count category (0.22 among participants with CD4 ≤ 250, 0.15 for 250 < CD4 ≤ 500, and 0.15 for CD4 > 500 cells/mm^3^, p < 0.01) but within each CD4 count category, no difference in median MLR was observed between participants with positive and negative TSTs (Fig 2). The violin plots suggest that the distribution of MLR may differ between CD4 count groups (Fig 2). The most striking difference in distribution of the probability density of the MLR occurred among TST negative participants with a low CD4 count (≤ 250 cells/mm^3^), for whom there was a broader range of MLR values compared to the higher CD4 count categories (p < 0.01). Visually, there was also a slight difference in distribution of the MLR probability density among TST positive participants with higher CD4 counts between 250 and 500 cells/mm^3^ or > 500 cells/mm^3^ (Fig 2). In stratified logistic regression analysis by CD4 count category, MLR was not associated with TST status in any CD4 category [aOR 0.95 (95% CI 0.30, 2.87) for CD4 ≤ 250, aOR 0.87 (95% CI 0.47, 1.72) for 250 < CD4 ≤ 500, aOR 1.35 (95% CI 0.94, 2.23) for CD4 > 500]. Spline regression did not change the findings to those obtained by standard logistic regression and did not result in a better model fit.

**Fig 3:**
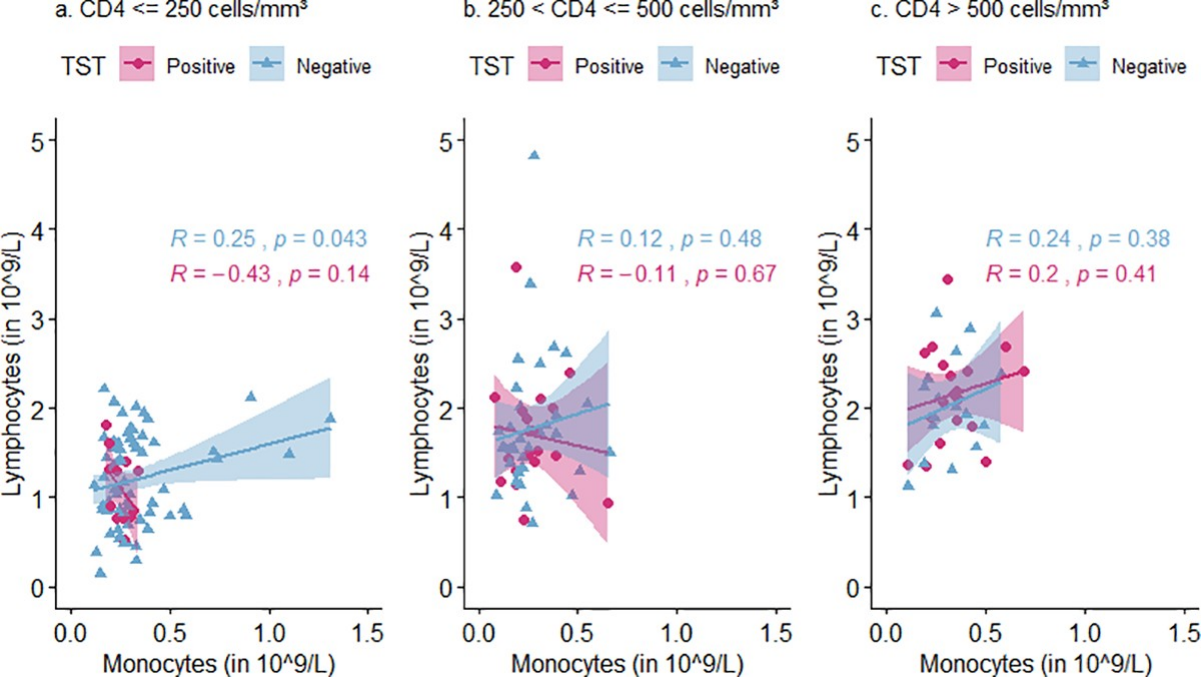
Relation between monocyte and lymphocyte count stratified by CD4 count category (≤ 250, 250 < CD4 ≤ 500, CD4 > 500) and TST status. For each CD4 count category, Pearson correlation coefficients (R) are calculated for those with a positive TST (displayed by pink dots) and those with a negative TST (displayed by blue triangles). TST = tuberculin skin test.

Monocyte and lymphocyte count correlated positively among participants with a negative TST in all CD4 count categories (Fig 3), but this was only significant in those with CD4 ≤ 250 (Pearson r = 0.25, p = 0.04, Fig 3A). Among TST positive participants, monocyte and lymphocyte count correlated positively in those with CD4 > 500 (Pearson r = 0.2, p = 0.41, Fig 3C) and negatively in those with lower CD4 [250 < CD4 ≤ 500 (Pearson r = -0.11, p = 0.67, Fig 3B) and CD4 ≤ 250 (Pearson r = -0.43, p = 0.14, Fig 3A)], but none of these correlations were significant.

## Discussion

Targeting individuals with *M*. *tuberculosis* infection who are at highest risk of progressing to active TB disease would greatly increase the risk-benefit balance of tuberculosis preventive therapy (TPT). Unfortunately, both the TST and IGRA markers of *M*. *tuberculosis* infection pose important logistic, cost and human resource barriers to such a targeted TPT strategy, particularly in low- and middle-income countries. mRNA expression signatures are being explored as alternatives to TST or IGRA-guided TPT, but these will most likely be costly and require sophisticated laboratory equipment not available in high TB burden countries [13,14]. The MLR could be a cheap alternative to the TST or IGRA in low income high TB burden settings, but our data unfortunately showed that the MLR was not independently associated with TST status, even within CD4 count categories. Even though we failed to observe an association between MLR and TST in this study, it remains possible that MLR can predict progression to active TB given that the TST cannot accurately identify people at high risk of progression to active TB disease.

It has been hypothesized that the MLR echoes immunity disturbances prognostic for adverse disease outcomes in malaria, influenza and TB [8,15,16]. With regards to TB, alterations in the relative frequency of monocytes to lymphocytes may affect an individual’s capacity to mount an effective immune response to mycobacterial infections or reflect (early) changes during increased bacterial replication and progression to from infection to TB disease [8,13,17–19]. In the early 1920s, Doan and Sabin´s reported that experimentally changing the MLR in rabbits was associated with higher lethality of *M*. *bovis* infection [20].

Our hypothesis that MLR is associated with TST status and thus the idea to use MLR to identify people at high risk of progression from *M*. *tuberculosis* infection to active TB was based on the findings of several clinical studies. HIV-infection or HIV disease progression increased MLRs and prolonged ART treatment have been shown to be associated with a return of MLRs to within the range observed for HIV-uninfected individuals [8]. A study in Italy showed that HIV-uninfected individuals with active TB disease had a higher MLR and a Chinese study showed that extremes in MLR (MLR < 9% or > 25%) were significant predictors of active TB [21,22]. Similarly, a prospective cohort of 1862 ART-naïve immunosuppressed (CD4 counts < 200 cells/mm^3^) individuals in South Africa reported that those with an MLR < 5^th^ or > 95^th^ percentile had a higher hazard (aHR 2.47; 95% CI, 1.39, 4.40) for development of TB during the first 5 years of ART [8]. Furthermore, prospective cohort studies among 296 HIV-uninfected TB contacts, 1202 HIV-infected post-partum women with higher CD4s > 250, and 1,336 South African infants (40% HIV-infected, 60% HIV-exposed and uninfected) observed that an elevated MLR was associated with increased risk of TB disease in the subsequent two years [8,15,23]. Finally, TB therapy significantly reduced MLRs in both HIV-infected and HIV-uninfected individuals [8,21,22].

Given the emerging data that MLR may identify people at higher risk of progression to active TB, we explored whether MLR could replace the use of a TST to identify people at high risk of development of active TB in people living with HIV in high TB burden countries. The findings of our study demonstrate that the MLR is unlikely to be a good alternative to the TST, as the median MLR was similar between TST positive and TST negative individuals, and the distribution almost similar. The only difference noted was an alteration of the MLR probability density among TST negative individuals with a low CD4 count ≤ 250 cells per mm^3^. This increased probability of higher MLR in negative TST patients with low CD4 count unlikely reflects a lowered risk of TB, but rather signals impaired HIV immunosuppression, which in turn is associated with a diminished hypersensitivity response to tuberculin [24,25]. We also noted a slightly different MLR distribution among TST positive people with higher CD4 count (> 250 cells/mm^3^), but could not confirm our hypothesis based on the findings of Naranbhai et al. that either a very high or very low MLR would be associated with TST positivity as a marker of TB risk.

The conflicting findings between associations observed between MLR and subsequent risk of developing active TB in prior studies on the one hand and the lack of association between MLR and TST positivity in our study on the other hand could be due to the fact that TST positivity is often the result of a exposure in the (distant) past whereas MLR may reflect changes in subclinical TB [26]. As such, both tests likely represent different correlates of TB risk along the causal pathway from exposure and infection to disease. A study from Madagascar in HIV-negative asymptomatic contacts of a TB case suggested that a combination of monocytes count and TST may improve the prediction of TB risk compared to TST or MLR alone, but this has not yet been replicated nor assessed in people living with HIV [23].

Our study was the first to evaluate the relation between MLR and TST, but the findings should be interpreted in the light of some limitations. Firstly, a quarter of eligible participants failed to return for TST reading and 10% did not have a valid laboratory result. While this might have led to selection bias, demographic and clinical characteristics did not differ between those excluded and those included in the analysis. Secondly, we designed the study as a cross-sectional analysis to assess the association between TST and MLR. As such, we were not able to assess a possible difference in the accuracy between MLR and TST in predicting the development of TB in the near future. We also did not assess the relationship between MLR and IGRA. Thirdly, because MLR can be dysregulated in a range of other infectious and non-infectious illnesses, we cannot exclude the influence of any underlying co-infection or other co-morbidity on the MLR results [16,27].

**In conclusion,** the findings of our study do not support our hypothesis that the MLR is associated with TST and may be an alternative for the TST. As neither TST or IGRA can accurately identify people living with HIV that are infected with *M*. *tuberculosis*, further research is needed to develop a cheap, simple and robust biomarker for targeting tuberculosis preventive therapy among people living with HIV.

## Supporting information

S1 Table Association between patient characteristics and TST positivity in 171 ART-naïve adults presenting for HIV care at a primary clinic in northern Johannesburg. (DOCX)
