# Supplementary material for: Association between monocyte-to-lymphocyte ratio and tuberculin skin test positivity in HIV-positive adults
Source: PLoS One. 2021 Jul 16;16(7):e0253907. doi: 10.1371/journal.pone.0253907 (PMC8284817; doi:10.1371/journal.pone.0253907)
Supplement: S1 Table — (DOCX) [file pone.0253907.s001.docx]

**Appendix,** **S1 Table.** **Association between patient characteristics and TST positivity in 171 ART-naïve adults presenting for HIV care at a primary clinic in northern Johannesburg.**

| Characteristic |  | TST positive (n=51) | TST negative (n=120) | OR for TST positivity (CI 95%) |
| --- | --- | --- | --- | --- |
| Age in years* |  | median 33  (IQR 29-39) | median 34  (IQR 28-40) | 1.01 (0.98, 1.05) |
| Gender | Male | 17 | 37 | Ref |
|  | Female | 34 | 83 | 0.89 (0.45, 1.82) |
| Pregnancy status | Not Pregnant | 43 | 110 | Ref |
|  | Pregnant | 8 | 10 | 2.05 (0.74, 5.54) |
| BMI in kg/m²*^#^*^,^* |  | median 25  (IQR 23-30) | median 25  (IQR 22-29) | 1.03 (0.98, 1.09) |
| History of TB treatment | No | 49 | 117 | Ref |
|  | Yes | 2 | 3 | 1.59 (0.20, 9.89) |
| HIV diagnosis (days from enrolment) | Diagnosed (≥ 1 days) | 5 | 10 | Ref |
|  | Newly diagnosed (< 1 days) | 46 | 110 | 0.84 (0.28, 2.81) |
| Most recent CD4 in cells/mm³* |  | median 354 (IQR 252-646) | median 233 (IQR 120-372) | 3.82 (1.89, 8.14) |
| CD4 count categories in cells/mm³* |  |  |  |  |
|  | CD4 ≤ 250 (n= 81) | 13 | 68 | Ref |
|  | 250 < CD4 ≤ 500 (n=54) | 18 | 36 | 2.62 (1.16, 6.04) |
|  | CD4 > 500 (n=36) | 20 | 16 | 6.54 (2.75, 16.28) |
| Monocytes in 10^9^/L* |  | median 0.27 (IQR 0.21-0.35) | median 0.26 (IQR 0.21-0.35) | 0.54 (0.05, 3.92) |
| Lymphocytes in 10^9^/L* |  | median 1.61 (IQR 1.30-2.14) | median 1.50 (IQR 1.00-1.80) | 1.68 (1.04, 2.77) |
| MLR* |  | median 0.17 (IQR 0.12-0.22) | median 0.18 (IQR 0.13-0.31) | 0.78 (0.59, 0.97) |

*All continuous variables are per one unit change, except for MLR (per 0.1 unit change) ^#^BMI data missing in 1 participant with positive TST. TST = tuberculin skin test; ART = antiretroviral treatment; HIV= human immunodeficiency virus; OR = odds ratio; CI = confidence interval, BMI = body mass index; TB= tuberculosis; MLR = monocyte-to-lymphocyte ratio
